# Transgenic Animals in Pharmaceutical and Biological Research in Iran

**Published:** 2013

**Authors:** Mehrdad Faizi

Transgenic (Tg) mice are very important models of disease, and have been introduced to biological studies since 1982. They are used for understanding the pathobiology of different diseases, finding targets for pharmacological manipulations, and for the evaluation of efficacy and toxicity of medicines in preclinical studies. Many different types of Tg mice are available as models for human diseases. For examples, there are Tg mice for different types of cancers, Down syndrome, Alzheimer’s disease, Huntington›s disease, diabetes mellitus, heart disease, hypertension, epilepsy, obesity, osteoporosis, glaucoma, blindness, deafness, anxiety, depression, and many other diseases.

Transgenic animals are used in toxicity assessments as well. There are several Tg animal models for mutagenicity assays approved by the World Health Organization like lacZTg mice for evaluation of mutagenicity of medicines. Tg-rasH2 mouse is another Tg model sensitive to both genotoxic and non-genotoxic carcinogens and is used in pharmaceutical industries as a substitute method to evaluate the carcinogenicity of novel compounds.

In recent years the global attention to biotechnological products has been dramatically increased. Transgenic animals can also be used to produce recombinant proteins.There are many examples of such applications for Tg animals. Productions of tissue plasminogen activator and recombinant human antithrombin III in goat are cases of using Tg animals in the biotech industries. Production of Factors VIII and IX and protein C in cows, or developing sheep milk includes thrombin and Factor XIII, and alpha-1-antitrypsin, or expression of malaria protein in mice for vaccine development are important examples for this kind of application.

The ethical concerns about the genetic modifications of animals are very important issues and have been closely debated in many scientific discussions.

In the last decade the contribution of Iranians in science production and their technological achievements, especially in medical and biological sciences, have been considerably increased. Therefore, demanding a greater consideration for genetically modified animals. Several strains of Tg mice are commercially available in many countries. However, the access to these animals for pharmaceutical researchers is very limited at this time in Iran. This requires serious attention from decision makers in the medical researchers in Iran in order for this problem to be solved. Commercially available Tg animals can be provided either by domestic or international private companies and will be a great service for scientists and researchers in Iran.

*Mehrdad Faizi* *is currently working as Associate Professor and Head of* *Department of Pharmacology and Toxicology, School of Pharmacy, Shahid Beheshti University of Medical Sciences,* *Tehran, Iran. He could be reached at the following e-mail address:* m.faizi@sbmu.ac.ir *or faizi64@ yahoo.com*

